# Drivers of medical spending behaviour amid the COVID-19 pandemic: Heuristic or systematic

**DOI:** 10.1016/j.rcsop.2022.100116

**Published:** 2022-02-07

**Authors:** Truc Nha Thi Phan, Vu Minh Ngo, Huan Huu Nguyen

**Affiliations:** a Institute of Business Research, University of Economics, Ho Chi Minh City 59C Nguyen Dinh Chieu street, Ward 6, District 3, Ho Chi Minh city, Viet Nam; b RMIT Unviersity, Vietnam 702 Nguyen Van Linh, District 7, Ho Chi Minh City, Viet Nam; c University of Economics Ho Chi Minh City, School of Banking, 59C Nguyen Dinh Chieu street, Ward 6, District 3, Ho Chi Minh city, Viet Nam

**Keywords:** Heuristic-systematic processes, Health-seeking behaviour, Perceived risk, Panic buying, Health belief model, COVID-19 pandemic

## Abstract

**Background:**

The COVID-19 pandemic has been creating unprecedented chaos and it could forever alter the way people live and work. Experiencing multiple waves of pandemic attacks could make people evolve their perceived risks about the health crisis, change their healthcare behaviours and medical spending to deal with the changing threats over time.

**Objectives:**

Even though there has been a great dealt of research on personal healthcare behaviours during the COVID-19 pandemic, the individual decision on medical spending has not been well explored. This study uses the health belief model and heuristic-systematic information processing theory to study the key drivers of medical spending behaviour as the COVID-19 pandemic evolved in Vietnam.

**Methods:**

Two surveys were conducted during the first (April 2020) and second waves (August 2020) of the COVID-19 pandemic resulted in a sample size of 1037 cases. The partial least square structural equation modeling (PLS-SEM) technique was employed to explore the structural relationships between health-seeking behaviours, pandemic perceived risks, panic buying, and demographic factors and how these sets of factors drive medical spending behaviours over time.

**Results:**

Comparing the two pandemic waves, this study finds significant distinctions in how people evaluate the risks of the pandemic and process information to make decisions about their medical spending. People were primarily influenced by the heuristic processes of panic buying patterns (β = 0.313, *p* < 0.001) and the health-related established habits in the first wave. Only in the second wave of the pandemic, the impact of the COVID-19 pandemic perceived risk has been recognized as a significant factor on medical spending via the comparison between perceived risks of the first and second pandemic waves (β = 0.262, *p* < 0.001).

**Conclusions:**

This study explores how individuals formulate their spending decisions in extreme conditions and provide valuable insights to help governments and institutions plan their policies to combat the COVID-19 pandemic more effectively.

## 1 Introduction

When WHO announced the pandemic of SAR-COV2 on 11 March 2020, the world faced harsh news: several dead people, the number of infected citizens, the plunging world economy, the rising unemployment rate, and the stock market turbulence the vaccine research process. The pandemic still does not end soon when infected cases continue to grow, and many countries experienced several waves of pandemic attacks. The new variation of SAR-COV2 has been detected in the United Kingdom in December 2020 and India in April 2021.^48^ Viet Nam is not an exceptional circumstance and also has suffered multiple waves of the COVID-19 pandemic attacks. As a result, experiencing multiple times of pandemic attacks makes people's health preventive behaviour evolved, likewise, health-seeking behaviour, perceived risk, and medical spending.

Research on personal healthcare behaviours and spending during the COVID-19 pandemic mostly focuses on the changes in individual healthcare cost and service use patterns. They found that people significantly reduced the use of preventive and elective care because of the social distancing measures and increased use of telemedicine.^17^^,^^23^^,^^43^ However, there is very little research on how individuals formulate their spending decisions on medical goods and services to cope with the COVID-19 pandemic. Regarding the unstable evolution of the pandemic over time in many countries, how the medical spending behaviours change and adapt to different COVID-19 pandemic waves need to be uncovered.

Taking the two waves of the COVID-19 pandemic attacks in April 2020 and August 2020 in Vietnam as a context, this study is based on the framework of the health belief model and the theory of heuristic-systematic information processing to investigate the decision-making method people might adopt for medical spending as the pandemic evolved. Specifically, individual perceived risks about the COVID-19 pandemic could hugely affect their decisions on medical spending. Additionally, for controlling individual heterogeneities related to individual healthy habits, we also would like to explore whether personal heal-seeking behaviours such as healthy lifestyle, resources, health spending, personal hygiene, mask-wearing, and risk avoidance^25^ could play an important role in forming the medical spending behaviours during the COVID-19 health crisis. In general, based on the health belief model, the primary goal of this study is to consider how personal medical spending behaviours have changed over time as the COVID-19 epidemic progresses and identify critical factors influencing these changes in medical spending behaviours. To interpret the paper's goal, specific research questions addressed in this study are constructed as follows: (1) How do individuals' health-seeking behaviours affect medical spending in the first and second waves of the COVID-19 pandemic? (2) How does the COVID-19 pandemic's perceived risk influence people's medical spending in the first and second waves of the COVID-19 pandemic? Moreover, (3) What are the differences in medical spending behaviours in the second wave of the COVID-19 pandemic compared to the first way?

The remained parts of the paper are constructed as follows. The following section discusses the theoretical framework for medical spending behaviour and its crucial determinants. The third part explains the study's methods and data, while section four delivers the findings. Lastly, the final section offers some study conclusions and further analysis.

## 2 Theoretical background

### 2.1 Health belief model

Health belief model (HBM) is one of the most widely recognized models which adapt behaviour sciences to health problems to formulate frameworks for health behaviours.^15^ The framework assumes that people are afraid of diseases and that health-related behaviours are closely associated with the degree of fear and anxiety perceived by individuals as a threat.^39^ In addition, the health-related behaviours should reduce the threat and the expected threat-reduction benefit should outweigh the obstacles to take actions.^15^ The three main sets of factors of the HBM framework include individual perceptions, modifying factors, and health-related behaviours as the dependent construct.^28^ The modifying factors consist of demographic variables, health-related knowledge and habits, and cues to action. The individual perceptions include perception of the health problems, perceived severity, and perceived susceptibility.^16^

**Fig. 1:**
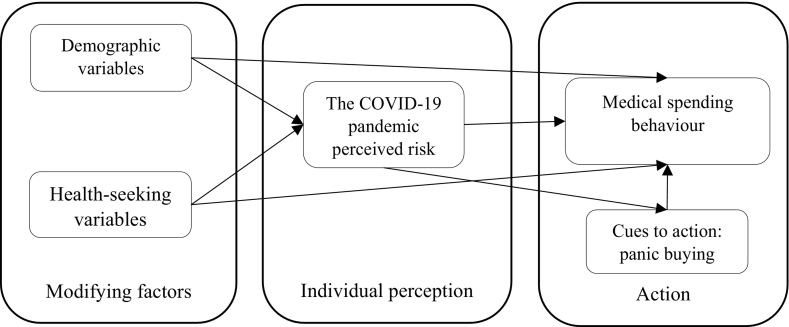
Conceptual research model adapted from HBM framework.

In this study, based on the HBM framework, we would like to investigate the drivers and motivations of individual medical spendings in the COVID-19 pandemic. As presented in Figure 1, the modifying factors include demographic variables (gender, income, education level, and age) and health-seeking behaviours variables (healthy lifestyle, resources spending, personal hygiene, mask-wearing, and risk avoidance). The individual perceptions include the COVID-19 pandemic perceived risk as a proxy for the perceived threat of the pandemic. Additionally, we include the panic buying variable as a cue to action factors to explore its role in motivating medical spending patterns. More importantly, because of the unprecedented scales and impacts of the Covid-19 pandemic on human life, this study proposes that the relative importance of each component in the HBM framework could significantly change over time. The heuristic-systematic information processing theory^8^ is employed to explores this phenomenon and how individual perception about the pandemic evolves and influences medical spending over time.

### 2.2 Heuristic-systematic information processing theory and panic buying

People need information on the COVID-19 pandemic to evaluate the threat of the pandemic and guide their healthcare behaviours such as medical spending. The heuristic-systematic processing posits two types of information processing: heuristic and systematic. Systematic information processing involves employing comprehensive attention, deep thought, and intensive reasoning to grasp information, whereas heuristic processing focuses on pertinent clues that help build good judgmental shortcuts.^8^ For example, Liberman and Eagly^26^ defined the levels of confidence gap as a difference between the expected levels of individual confidence to act and the actual confidence level which individuals get from available information. The wider the confidence gap is, the more heuristic processing employs and vice versa with the lesser confidence gap, individuals tend to use systematic information processing.

**Fig. 2:**
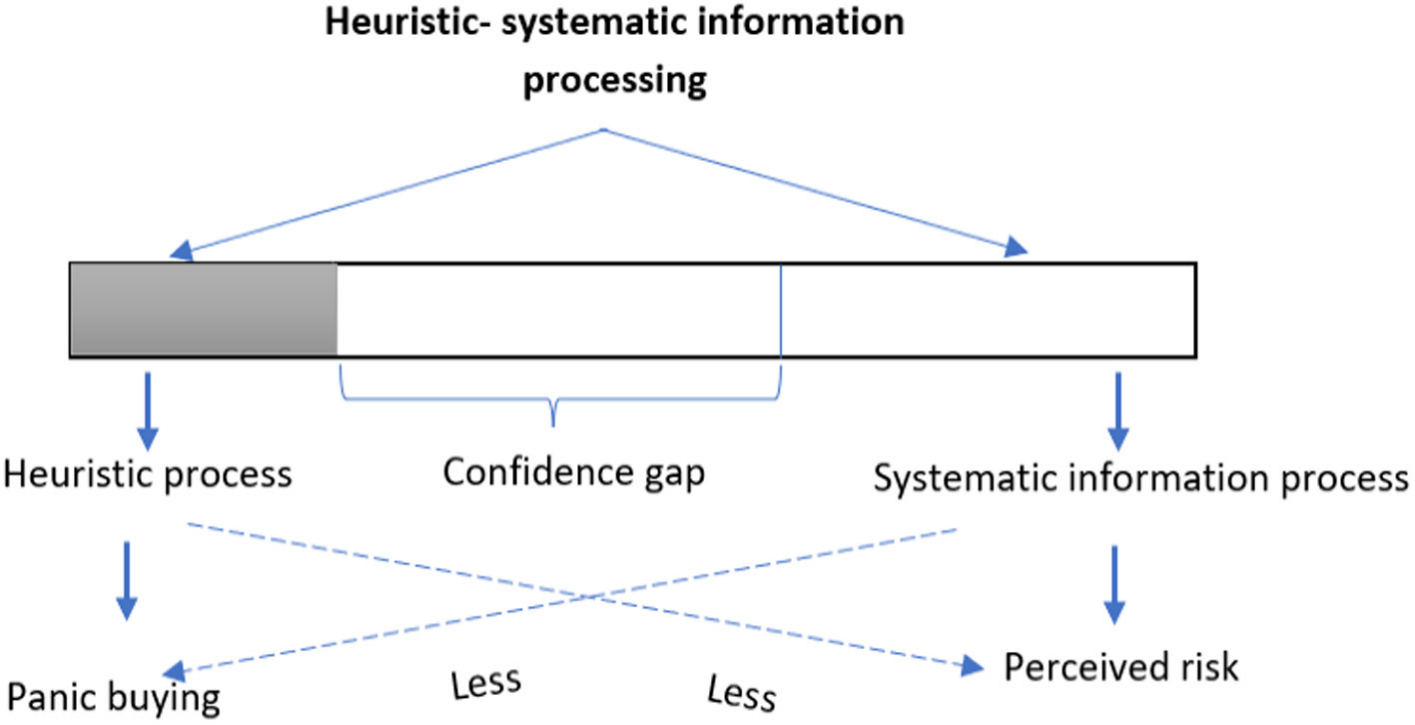
Heuristic-systematic information processing model as the COVID-19 pandemic evolves over time.

At the first time encountering a new situation, individuals tend to seek only enough information to help them make quick choices within their limited information-processing capability which contributes to a very large confidence gap in making an informed decision. In other words, they tend to develop heuristics or rules of thumb to make decisions when they are not confident in their insights extracted from processing limited available information.^15^ However, over time, the individual will obtain more information and use more cognitive effort to reach a particular level of confidence on a continuum of judgmental confidence (Figure 2). At this point, systematic processing will occur to replace the heuristic processing. For example, in the panic scenario at the beginning of the COVID-19 pandemic, heuristic processing is considered the life-vest since it can occur even when humans are not motivated or consciously think about a topic. People focus on quickly visible and quickly understood clues for decision making, such as a communicator's qualifications, the communicator's group membership, the number of arguments offered, or audience response is referred to as heuristic processing.^8^

Furthermore, as the COVID-19 pandemic continuously breaks out, people gain more pandemic-related information and experience, which can be seen as valuable inputs for their later information-processing and decision-making process. People often act in heuristic processing until they are both motivated and capable of deliberative thought. They are found to deviate from the heuristic model when they have enough information for their cognitive effort.^9^ For instance, a systematic approach is more viable considering the efforts to raise awareness of the personal hygiene, mask-wearing, and social distancing measures during the COVID-19 that the Vietnamese government deploys and constantly reminds through media, newspapers, newspapers, and the internet. Thus, in the later pandemic stage, a systematic approach to information processing could be the dominant model when people decide on their medical spending behaviours. Through meticulous attention, in-depth thought, and extensive reasoning, systematic processing attempts to comprehend all given information completely.^8^ This information is utilised to direct attitudes, judgments, and behaviour in later instances.

### 2.3 Health-seeking behaviour

The health-seeking behaviour construct consists of six health-related behaviours which are healthy lifestyle, resources health spending, personal hygiene, mask-wearing, and risk avoidance.^51^ In this study, health-seeking behaviours play the role of modifying factors in the HBM framework which is proposed to significantly impact the pandemic perceived risk and medical spending during the COVID-19 pandemic. Researchers have found interrelationships between health-seeking behaviours and the perceived risk of the COVID-19 pandemic.^40^^,^^50^^,^^51^ Some research found that the health crisis significantly affects individuals' health-seeking behaviours.^25^^,^^40^ Following this view, the pandemic could impact the individual's health habit by changing the individual perceptions on the health-related issues including the perception of threats of the health crisis or the importance of having good health behabiours.^25^

On the other hand, the other researchers found that the pre-COVID-19 health conditions and habits could have significant roles in forming their perceived risk of the pandemic.^50^^,^^51^ According to this view, the differences in health conditions and habits resulted from the individuals' health-seeking behaviours could impact their perception of the COVID-19 pandemic threats and also their actions to protect themselves.^50^ This study followed the latter view and based on the HBM framework to proposed that health-seeking behaviours could play vital roles in forming the perceived risks about the COVID-19 pandemic and, in turn, medical spending behaviours. Moreover, as the pandemic could also change the health-seeking behaviours in the later pandemic stages,^40^ this study also separately explores how this relationship performs in the two waves of the pandemic.

H1a Health-seeking behaviours are associated with the perceived risk of the COVID-19 pandemic in the first waves of the pandemic.

H1b Health-seeking behaviours associated with the perceived risk of the COVID-19 pandemic in the second waves of the pandemic.

H2a Health-seeking behaviour is significantly associated with the medical spending in the first wave of the COVID-19 pandemic.

H2b Health-seeking behaviour is significantly associated with the medical spending in the second wave of the COVID-19 pandemic.

### 2.4 The COVID-19 pandemic perceived risk, panic buying, and medical spending decision

Uncertainty causes people hard to make the decision, especially when they confront unexpected matters. The perceived risk theory contributed to justifying human behaviour when attempting to evade risk and maximise their expectations. In a health crisis, the realisation of risk help people get appropriate treatment, reduce the useless resource, and unproductive cure.^35^ The more risk individuals perceive the pandemic's effects on their health, and the more motivated they are to engage in prevention behaviours.^7^^,^^41^ According to Kahneman and Tversky,^22^ in prospect theory, humans are not rational when making decisions, and humans may assign an overly high decision weight for low probability events from the perception of risk. For example, no evidence shows that nutritious food can help people have robust health that fights against the pandemic and spend a lot on medical goods can help us avoid the COVID-19 pandemic. However, the loss-aversion tendency and perceived risk could motivate individuals to perform preventive behaviours, including increasing medical spending to deal with the health crisis. In this paper, the individual perceived risk of the COVID-19 pandemic is proposed to significantly affect the level of medical spending in both two waves of the pandemic.

H3a Perceived risk of the COVID-19 pandemic is significantly associated with the medical spending in the first wave of the COVID-19 pandemic.

H3b Perceived risk of the COVID-19 pandemic is significantly associated with the medical spending in the second wave of the COVID-19 pandemic.

However, Mitchell^31^ found that humans who have never experienced the situation cannot precisely measure the risk, which needs sufficient information and cognitive efforts. Covid19 was such a different epidemic to most people when they first encounter it. It evolved in a complicated pattern with very high dead rates, many times more than seasonal flu. Tversky and Kahneman^46^ point out that people conduct many heuristics to value the news. Thus, in this case, the availability heuristic could be used as a shortcut about the pandemic of Covid 19 when they first knew about the pandemic via news and media.

Nonetheless, using heuristics might run to erroneous choices, such as perception biases such as herd behaviours and panic buying as mentioned above.^8^ The easiest way to apply heuristic processing is by replicating other people's behaviours which could explain a close relationship between heuristic and herb behaviour. Dependence is readily apparent as people copy other people's behaviour instead of using their expertise. The concept of “herd” has long been debated in crowd psychology and behavioural finance. “Herd” means the large group of people mainly do the same thing at the same time,^3^ and its constitution is differentiated by feelings of dread, including anxiety.^6^^,^^12^ Some examples of herd behaviour in the stock market are the rise of noise traders, especially the stock market bubbles.^3^ Herd behaviours explain the panic buying behaviours observed globally at the beginning of the COVID-19 pandemic across the world.^10^^,^^30^^,^^34^ The uncertainty, perceptions of severity, and anxiety triggered by the COVID-19 pandemic could be the main reasons for herb behaviours and panic buying in the early stage of the pandemic.^34^ The number of latex gloves, masks did not go far enough for the surgeons, nurses and those on the front lines of the outbreak. People were warier of the threat's significance when they discovered that the medical supplies were being shortage due to the pandemic. This study proposes that people have an astounding propensity for impulsive conduct at the beginning of the pandemic, as shown by the high consumption of medical supplies during the first wave. In this study, we are keen to explore the effects of panic buying on the decision of medical spending as the pandemic progressed and proposed that the more panicked people are in their buying behaviours, the more medical spending is. As such, panic buying is proposed in this study to mediate the effects between the perceived risk of the COVID-19 pandemic and the medical spending behaviours as follows.

H4 Panic buying behaviour mediates the relationship between the perceived risk of the COVID-19 pandemic and the medical spending in the first wave of the COVID-19 pandemic.

Vietnam promptly managed the spread of the Covid19 virus using different rigid actions to relieve the harmful impacts. Wearing a mask, social distancing, living quarter hygienic, maintaining good personal hygiene, and frequently washing hands are typically successful communication campaigns at pivotal points of the epidemic's progression. As a result, Vietnam successfully contained the first wave of the pandemic very quickly. When the second wave broke out, it was expected that Vietnamese citizens have enough information and experience to form more precise risks about the pandemic and inform their related preventive decisions, including medical spending. In the second wave, individuals tend to use more of their cognitive perception as a systematic information process, and panic buying seems to have a more negligible effect on medical spending.

Moreover, in the second stage of the pandemic, it may be no doubt that Vietnamese people might accumulate knowledge for using heuristic from the previous pandemic wave. Thus, the perceived risk of the pandemic in the second wave of the pandemic could vary widely and depend on individual perception and experience from the first wave.^37^^,^^44^ In this case, the change in perceived risk of the pandemic between the first and second waves could be a determinant factor affecting the next course of preventive behaviours. As a result, in wave two, it can be presumed that the mediating impact of panic buying is insignificant.

H5 The differences in COVID-19 pandemic perceived risk between the first and second wave mediate the relationship between COVID-19 pandemic perceived risk and the medical spending in the second wave of the COVID-19 pandemic.

## 3 Methodology

### 3.1 Data and sample

This study aims to explore the impacts of health-seeking behaviours on medical spendings in the COVID-19 pandemic. The purposive sampling method collects data via two online surveys in April 2020 and August 2020.^13^ Given the semi-experimental nature of this research, respondents' access and willingness are prioritised to maximise the likelihood of participating in both surveys in wave and wave 2 of the COVID-19 pandemic in Vietnam. Targeted respondents also need to reside in cities where the risks of virus infections are considerable, and access to medical goods/services are available (i.e., Ho Chi Minh, Hanoi, Da Nang). A filter question is included to ensure respondents met this residency criterion.

Because of the social distancing measures, online channels via email and webpages are employed to distribute questionnaires. In addition, lottery incentives increase the response rate and acquire more contact information.^24^^,^^52^ The first survey in April 2020 yield 1575 respondents. We contacted the respondents who participated in the first survey in August via email and phone (if possible) to solicit their participation in the second survey. As a result, a sample of 1055 completed responses to both surveys is collected. After removing missing data and suspicious responses, the final sample consists of 1037 respondents who completed both surveys in April and August 2020.

We follow Lau et al.^25^ to treat health-seeking behaviours in an influenza pandemic context as a combination of different types of health-related behaviours, including health services seeking, healthy lifestyle, resources spending on health, personal hygiene, mask-wearing, and risk avoidance. All these health-related behaviour constructs are measured using reflective models of multiple items. All questionnaire items are adapted from previous empirical studies and are operationalised using the 7-point Likert scale (1: strongly disagree, 7: strongly agree) and the reflective model.

Regarding the perceived risk of the COVID-19 pandemic, we base on the work from Wilson et al.,^49^ which advocated the use of a multidimensional scale to measure the perception of risks regarding both the magnitude and probability of the risky event. This approach of measuring risk perception could capture the individual assessments of whether to take protective actions or not based on the severity of consequences and the emotional reactions to the events.^49^ Additionally, a variable using a five-point scale (1 – significant decrease, 2- decrease, 3 – no change, 4 – increase, 5- significant increase) is also included to measure the changes in respondents' perceived risk of the COVID-19 pandemic between the first and second wave of the pandemic in Vietnam.

Several scales are developed for panic buying in the COVID-19 pandemic ^5^. In this paper, we adopt the approach of Bentall et al. ^5^ to measure the increase in spending on food and other necessary goods due to the panic buying behaviours using the five-point scale (1 – not at all, 2 – very slightly, 3- moderately, 4 - to a significant degree, and 5 - very significant). Furthermore, the same approach is used to operationalise the changes in medical spending construct in wave one and wave two of the pandemic. Finally, the respondents' demographic information questions are also included in the questionnaire.

Items used in the questionnaire are translated into Vietnamese from English using the back-translation method. A pilot test of the questionnaire is also conducted on 25 respondents. Two professors review the pilot test's healthcare and risk management results to ensure content validity and appropriateness in the context of Vietnam. Minor adjustments were made base on their feedbacks. For example, questions of resources spent on health are accompanied by examples of what types of resources are usually considered to reduce ambiguity.

### 3.2 Analysis methods

This study chooses the variance-based partial least squares structural equation modeling (PLS-SEM) method over the covariance-based one (CB-SEM) to test the proposed hypotheses. The first benefit of PLS-SEM is that the bootstrapping procedure reduces estimation bias significantly as there are no strict assumptions of data distribution of the constructs as in CB-SEM.^18^ Also, PLS-SEM allows the test of multiple mediators simultaneously and can provide the statistical test for the significance of total indirect effects of combined mediating effects.^20^ Furthermore, PLS-SEM is preferred when estimating exploratory and highly complex models.^42^ SmartPLS3 software^38^ is used for statistical estimation of the structural equation model, and the results are presented following guidelines suggested by.^19^ First, the reliability and validity of measurement instruments are verified using measurement model estimation. Then, in the second stage, the structural model consisting of interrelationships between constructs is examined to test the proposed hypotheses.

## 4 Data analysis and results

Table 1 provides demographic information about respondents, showing more males (59.6%) than females (40.8%) participating in both surveys. More than half of the respondents are between 18 and 35 (52.5%) and have a monthly income between 10 and 20 million VND (53.2%). More than 40% of the respondents significantly increased their medical spending in the 1st wave of the COVID-19 pandemic. The proportion changed considerably in the 2nd wave when most respondents increased their medical spending level very slightly or moderately (~61%). Regarding the change in perceived risk between the 1st and second waves, more than 70% of respondents perceived the 2nd wave was more dangerous to them than the 1st wave. About 23% of respondents claimed that the risk level was the same between the two waves. The demographic data of respondents (age, income level, education level, gender) are used as control variables in the structural model controlling for the impacts of respondent's background on factors of interest.

**Table 1:** Respondent Profile.

Profile	n	%	Profile	n	%
Gender			Change in medical spending in Wave 1		
Male	614	59.6%	Not at all	209	20.2%
Female	423	40.8%	Very slightly	97	9.4%
Age			Moderately	248	23.9%
18–35	544	52.5%	To a significant degree	424	40.9%
35–45	337	32.5%	Very significant	59	5.7%
45–60	144	13.9%	Change in medical spending in Wave 2		
above 60	12	1.2%	Not at all	301	38.7%
Marital status			Very slightly	369	35.6%
married	448	43.2%	Moderately	266	16.0%
single	589	56.8%	To a significant degree	64	6.2%
Income_level (million VND/month)			Very significant	37	3.6%
less than 10	29	2.8%	Perceived risk Wave1 vs Wave2		
10–20	552	53.2%	Significant decrease	23	2.2%
20–30	393	37.9%	Decrease	40	3.9%
30–45	50	4.8%	No change	241	23.2%
above 45	13	1.3%	Increase	463	44.6%
Education level			Significant increase	270	26.0%
Diploma or lower	8	0.07%			
Bachelor's degree	943	90.9%			
Postgraduate degree	86	8.2%			

### 4.1 Common method bias

Common method bias (CBM) could concern the model's validity by using self-administered questionnaires to collect data. Therefore, we follow the approach from Podsakoff and Todor^36^ and produce an unmeasured marker variable (UMV) to account for the CBM using unrotated factor analysis. First, the factor analysis is executed using SPPS software, and the factor score of the first unrotated factors is used as data for UMV. Then, the UMV is included as an exogenous variable in the structural model to explain all the endogenous variables. Finally, the R2 in the new model is compared to the original model (without UMV). The result is shown in Table 2, indicating that the presence of UMV does not lead to significant changes in R2 in all endogenous variables. Thus, CRM is not a significant issue to the findings' validity in this paper.

**Table 2:** Assessment of common method variance.

Endogeneous	Wave 1			Wave 2		
	R^2^	R^2^(with UMV)	Differences	R^2^	R^2^ (With UMV)	Differences
Change in medical spending	0.131	0.134	0.003	0.148	0.151	0.003
Panic buying	0.011	0.012	0.001	0.012	0.013	0.001
Pandemic perceived risk	0.075	0.077	0.002	0.079	0.081	0.002
Perceived risk Wave1vs2				0.069	0.072	0.003

### 4.2 Measurement model

The measurement model is assessed to assure the validity and reliability of the research model's constructs for both research models in wave one and wave two of the pandemic. The convergent validity and reliability are first examined utilising internal consistency. Table 3 indicates that 17 of 20 indicators have outer loadings more significant than 0.7, and 3 loadings are between 0.6 and 0.7.^20^ Furthermore, the constructs' average variance extracted (AVE) are more significant than the recommended threshold of 0.5.^14^ These results support the constructs' convergent validity. All Cronbach's α and composite reliability (CR) values exceed the level of 0.7 suggested by.^20^ Thus, constructs' internal reliability could be established in this research.

**Table 3:** Measurement model.

		Wave 1				Wave 2			
		Convergent validity		Internal reliability		Convergent validity		Internal reliability	
	Indicators	Loadings	AVE	α	CR	Loadings	AVE	α	CR
Healthy lifestyle	HL1	0.698	0.778	0.881	0.905	0.883	0.781	0.91	0.905
	HL2	0.801				0.862			
	HL3	0.808				0.786			
	HL4	0.819				0.783			
	HL5	0.838				0.688			
	HL6	0.705				0.674			
	HL7	0.626				0.620			
Health resources spending	HR1	0.849	0.817	0.791	0.899	0.943	0.887	0.876	0.84
	HR2	0.955				0.931			
Health service seeking	HSS1	0.948	0.875	0.863	0.933	0.948	0.923	0.919	0.96
	HSS2	0.973				0.973			
Personal hygiene	PHG1	0.867	0.792	0.869	0.92	0.866	0.826	0.897	0.934
	PHG2	0.918				0.940			
	PHG3	0.885				0.919			
Mask wearing	MW1	0.939	0.897	0.885	0.946	0.943	0.878	0.861	0.935
	MW2	0.955				0.931			
Panic buying	PB1	0.734	0.734	0.835	0.861	0.768	0.721	0.813	0.874
	PB2	0.823				0.876			
Pandemic perceived risk	PR1	0.812	0.732	0.827	0.842	0.877	0.759	0.836	0.873
	PR2	0.853				0.834			
Perceived risk Wave1vs2	PR1vs2					1.000	1.000	1.000	1.000

Discriminant validity is tested using the Heterotrait-Monotrait (HTMT) ratio of correlations. The more conservative fixed cut-off value of 0.75 suggested by^47^ is used in this study as the criterion for examining the HTMT correlations between constructs. The nearer the HTMT correlation value is to 1.0, the more likely the discriminant validity is violated. Results from Table 4 indicate that all HTTM values in both models, wave 1 (Panel A) and wave 2 (Panel B), are less than 0.75 except for one value between constructs Mask wearing and Personal hygiene (0.773) in Panel B. The second way of using HTTM correlations includes the null hypothesis test (H0: HTMT ≥ 1). If the H0 holds, then the discriminant validity is violated. The confident interval analysis presented in Table 4 rejects the null hypothesis indicating that the discriminant validity of all constructs in both waves is supported.

**Table 4:** Assessment of discriminant validity using the Heterotrait-Monotrait ratio of correlations criterion (HTMT _0.85_ criterion).

	HL	HR	HSS	PHG	MW	PB
Panel A- Wave 1						
HR	0.638[0.57–0.69]					
HSS	0.666[0.61–0.71]	0.62[0.55–0.6]				
PHG	0.664[0.58–0.69]	0.544[0.48–0.59]	0.649[0.59–0.70]			
MW	0.547[0.48–0.60]	0.435[0.36–0.49]	0.568[0.50–0.62]	0.745[0.68–0.79]		
PB	0.563[0.51–0.62]	0.540[0.50–0.57]	0.547[0.50–0.61]	0.539[0.50–0.57]	0.531[0.50–0.58]	
PR	0.547[0.51–0.55]	0.537[0.50–0.54]	0.608[0.58–0.67]	0.636[0.57–0.69]	0.605[0.58–0.65]	0.638[0.60–0.68]


Panel B- Wave 2						
HR	0.669[0.61–0.71]					
HSS	0.689[0.64–0.73]	0.593[0.53–0.63]				
PHG	0.689[0.63–0.72]	0.508[0.44–0.55]	0.683[0.63–0.72]			
MW	0.612[0.54–0.66]	0.429[0.36–0.49]	0.686[0.63–0.73]	0.773[0.72–0.81]		
PB	0.595[0.53–0.65]	0.432[0.36–0.51]	0.591[0.52–0.65]	0.447[0.40–0.59]	0.571[0.51–0.63]	
PR	0.550[0.51–0.65]	0.557[0.51–0.63]	0.563[0.51–0.61]	0.666[0.60–0.73]	0.678[0.59–0.74]	0.564[0.51–0.62]

Note HL-Healthy lifestyle; HSS-Health service seeking; PHG-Personal hygiene; MV-Mask Wearing; PB-Panic buying; PR- Pandemic Perceived risk. The bracket is the lower and upper value of 95% confident interval of HTMT correlations using bootstrapping.

### 4.3 Structural model

First, we examine the collinearity between constructs in the structural model. Results show that all exogeneous variables' variance inflation factor (VIF) values are below the recommended threshold of 3.3.^11^ Next, direct and indirect relationships between constructs are evaluated. Finally, a 5000-iterations procedure of the bootstrapping is executed to estimate the 95% bias-corrected confidence interval for the structural paths of interest. Results are presented in Table 5 for wave 1 (Panel A) and wave 2 (Panel B). In the model of wave 2, medical spending in wave one is added as control variables accounting for the previous level of spending, which are expected to affect the level of spending in wave two.

**Table 5:** Structural model testing.

Paths		Panel A: Wave 1				Panel B: Wave 2			
		β	t-value	*p*-value	95 BCE% Confident interval	β	t-value	p-value	95 BCE% Confident interval
Direct effects									
a_11_	HL → PR	−0.058	1.176	0.24	[−0.16,0.02]	−0.115^⁎^	2.030	0.042	[−0.23, −0.04]
a_12_	HL → MS	−0.100^⁎^	2.277	0.023	[−0.18, −0.01]	−0.009	0.199	0.843	[−0.08,0.09]
a_21_	HR → PR	−0.049	1.077	0.282	[−0.14,0.02]	−0.082^⁎^	2.021	0.045	[−0.15, −0.00]
a_22_	HR → MS	0.103^⁎^	2.079	0.038	[0.01,0.17]	−0.017	0.398	0.691	[−0.04,0.11]
a_31_	PH → PR	0.132^⁎⁎^	2.806	0.005	[0.05,0.23]	0.174^⁎⁎⁎^	3.496	<0.001	[0.08,0.26]
a_32_	PH → MS	−0.123^⁎^	2.691	0.007	[−0.21, −0.03]	−0.062	1.152	0.249	[−0.18,0.04]
a_41_	MW → PR	0.042	1.002	0.317	[−0.03,0.12]	0.126^⁎⁎^	2.787	0.005	[0.04,0.21]
a_42_	MW → MS	0.080^⁎^	2.338	0.045	[0.04,0.10]	−0.022	0.431	0.667	[−0.12,0.07]
a_51_	HSS → PR	0.103^⁎^	2.351	0.019	[0.02,0.18]	0.102^⁎^	2.041	0.041	[0.07,0.18]
a_52_	HSS → MS	0.009	0.213	0.832	[−0.07,0.08]	−0.017	0.398	0.691	[−0.11,0.06]
a_61_	RA → PR	−0.064	1.622	0.106	[−0.13,0.01]	−0.005	0.137	0.891	[−0.06,0.07]
a_62_	RA → MS	0.029	0.746	0.456	[−0.04,0.09]	0.085^⁎^	2.207	0.027	[0.01,0.15]
b_1_	PR → MS	0.062	1.823	0.077	[−0.00,0.12]	0.017	0.578	0.563	[−0.04,0.06]
b_2_	PR → PB	0.046^⁎^	2.292	0.041	[0.02,0.10]	0.065^⁎^	2.475	0.013	[0.01,0.11]
b_3_	PB → MS	0.313^⁎⁎⁎^	9.592	<0.001	[0.25,0.38]	−0.004	0.140	0.888	[−0.05,0.05]
b_4_	PR → PR1vs2					0.262^⁎⁎⁎^	6.572	<0.001	[0.18,0.34]
b_5_	PR1vs2 → MS					0.061^⁎^	2.118	0.034	[0.00,0.11]
c_1_	Gender → MS	0.022	0.698	0.485	[−0.04,0.08]	0.003	0.107	0.915	[−0.05,0.06]
c_2_	Income → MS	0.077^⁎^	2.195	0.048	[0.00,0.12]	−0.050	1.635	0.102	[−0.10,0.00]
c_3_	Age → MS	−0.014	0.434	0.664	[−0.07,0.05]	−0.015	0.529	0.597	[−0.06,0.04]
c_4_	Education → MS	−0.056	1.421	0.156	[−0.14,0.01]	−0.100^⁎⁎^	2.979	0.003	[−0.15, −0.03]
c_5_	MS1 → MS2					0.350^⁎⁎⁎^	10.86	<0.001	[0.28,0.41]

Indirect effects									
a_11_b_1_	HL → PR → MS	−0.004	0.881	0.378	[−0.01,0.00]	−0.003	0.588	0.557	[−0.01,0.00]
a_21_b_1_	HR → PR → MS	−0.003	0.883	0.378	[−0.01,0.00]	−0.001	0.467	0.641	[−0.00,0.00]
a_31_b_1_	PH → PR → MS	0.008	1.627	0.145	[−0.00,0.02]	0.003	0.571	0.569	[−0.00,0.01]
a_41_b_1_	MW → PR → MS	0.003	0.842	0.400	[−0.00,0.01]	0.002	0.548	0.584	[−0.00,0.01]
a_51_b_1_	HSS → PR → MS	0.006	1.655	0.148	[−0.00,0.02]	0.000	0.12	0.904	[−0.00,0.00]
a_61_b_1_	RA → PR → MS	−0.004	1.246	0.213	[−0.01,0.00]	0.000	0.07	0.944	[−0.00,0.00]
b_2_b_3_	PR → PB → MS	0.024^⁎^	2.121	0.042	[0.01,0.03]	0.000	0.136	0.892	[−0.01,0.00]
b_4_b_5_	PR → PR1vs2 → MS					0.016^⁎^	2.006	0.045	[0.00,0.03]

Note: BC - bias-corrected; Bootstrap based on n, 5000 resample (two-tailed); HL-Healthy lifestyle; HR- Health resource; HSS-Health service seeking; PHG-Personal hygiene; MV-Mask Wearing; RA- Risk avoidance; PB-Panic buying; PR- Pandemic Perceived risk; MS- change in medical spending; MS1 – Change in medical spending in Wave 1; MS2- Change in medical spending in Wave 2; PR1vs2 – change in perceived risk of the COVID-19 pandemic in wave two compared to wave one. ^⁎^*p* < 0.05; ^⁎⁎^*p* < 0.01; ^⁎⁎⁎^*p* < 0.001.

### 4.4 Health-seeking behaviours to pandemic perceived risk

**Fig. 3:**
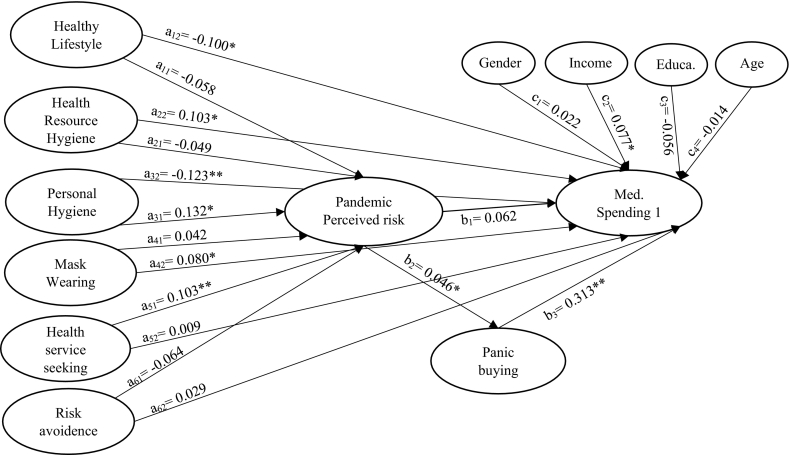
Health-seeking behaviours, perceived risks of the Covid-19 pandemic and medical spending in the 1st wave.

**Fig. 4:**
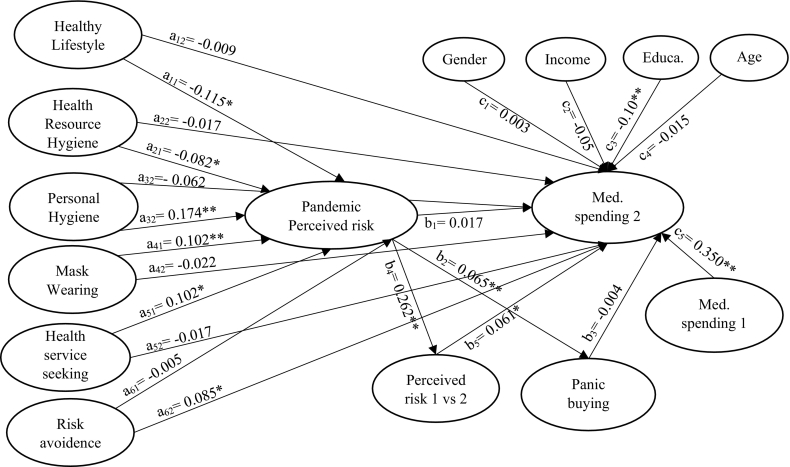
Health-seeking behaviours, perceived risks of the Covid-19 pandemic and medical spending in the 2nd wave.

Concerning the direct effects of health-seeking behaviours on the perceived risk of the COVID-19 pandemic, there are significant differences between wave one and wave two of the pandemic (Fig. 3, Fig. 4 and Table 5). In wave 1, direct paths from most health-seeking behaviours to perceived risk are not significant except for personal hygiene (β = 0.132, *p* < 0.01) and health service seeking (β = 0.103, *p* < 0.05). Whereas, in wave 2 model, direct paths from healthy lifestyle (β = 0.115, p < 0.05), health resources (β = −0.08, p < 0.05), personal hygiene (β = 0.17, p < 0.01), mask-wearing (β = 0.120, p < 0.01) are significant. There are significant changes in the impacts of health-seeking behaviours on the COVID-19 perceived risks between wave 1 and wave 2 of the pandemic. Notably, in wave 1, only health service-seeking behaviour which is associated with seeking health information and consults, are more likely to have significant impacts on risk perceptions about the COVID-19 pandemic than other health-seeking behaviours. This result emphasizes the fact that to formulate the proper pandemic risk perception, information processing capability is extremely important, especially at the beginning of the pandemic when related information is very limited. In wave 2, health resources spending behaviour is found to have negative impacts on the pandemic perceived risk. This result highlights that individuals who have spent a significant amount of time and financial resources on health issues regularly perceived the COVID-19 pandemic as less risky to them than those who spent fewer resources on health issues. In addition, given the high rate of infections via bad habits of hand hygiene, people with a higher standard of personal hygiene are much more cautious about the pandemic than those with a lower standard in both waves of the pandemic. Thus, both *H1a* and *H1b* are partially supported, but personal health-seeking behaviours generally have stronger effects on pandemic perceived risks in wave two than in wave one.

### 4.5 Health-seeking behaviours to medical spending

The opposite pattern between wave one and wave two can also be observed in the relationship between health-seeking behaviours and medical spending. In wave 1, most health-seeking behaviours including healthy lifestyle (β = −0.100, *p* < 0.05), health resource (β = 0.103, p < 0.05), personal hygiene (β = −0.123, p < 0.05), mask-wearing (β = 0.080, p < 0.05) significantly influence to medical spending (Figure 3). However, in wave two (Figure 4), these significant direct relationships between health-seeking behaviours and medical spending fade away and only risk avoidance (β = 0.085, *p* < 0.05), which significantly impact medical spending. Thus, *H2a* is strongly supported, and *H2b* is very weakly supported. Especially in wave 1, opposite signs of coefficients in these relationships can be observed. Specifically, a healthy lifestyle and personal hygiene negatively impact medical spending, while health resources and mask-wearing exert positive ones. According to these findings, people who currently have a healthy lifestyle or high standard of personal hygiene do not spend as much as those who currently have a less healthy lifestyle or lower standard of personal hygiene.

In general, in wave 1, respondents' medical spending is driven mainly by the direct effects of health behaviours and skip the risk perception process. In Wave 2, after controlling for the previous level of spending in wave one, the effects of health-seeking behaviours on spending behaviours in completely turnaround compare to wave 1. Instead, individual health-seeking behaviours now primarily contribute to the respondents' perception of spending behaviours in wave 2. This turnaround is consistent with the heuristic-systematic framework as a theoretical background for this paper.

### 4.6 Pandemic perceived risk to medical spending

In wave 1, the direct relationship between pandemic perceived risk and medical spending is positive but not significant (wave 1: β = 0.062, *p* = 0.077). Thus, *H3a* is not supported in this model. In urgent and stressful events like the COVID-19 pandemic, as suggested by the heuristic framework of information processing, a shortcut to formulate decisions is preferred to rationally analyse risks that need enough information and resources for cognitive processes. Therefore, in this paper, panic buying is proposed to dominate and mediating the relationship between pandemic perceived risk and medical spending in wave 1.

### 4.7 Mediating effects of panic buying

In wave 1, the panic buying construct is proved to have significant mediating effects for the relationship from perceived risk to medical spending (β = 0.024, *p* < 0.05). Significantly, the direct impact of panic buying is one of the most potent effects on medical spending in wave 1 (β = 0.313, p < 0.05). As suggested by the path of perceived risk → panic buying → medical spending, individuals who perceived the COVID-19 pandemic as riskier tend to be subject to panic buying behaviour and, in turn, lead to higher spending on medical goods/services in wave 1. Thus, *H4* is supported.

### 4.8 Mediating effects of changes in pandemic perceived risk

In contrast with the model in wave1, panic buying lost its effects on medical spending in wave 2 (β = −0.004, *p* = 0.888). Instead, the mediating effect of changes in perceived risk between the two waves of pandemic (PR1vs 2) is significant (β = 0.016, *p* < 0.05) in the model of wave 2. The changes in perceived risk fully mediate the relationship between perceived risk and medical spending, given that the direct path of this relationship is insignificant (β = 0.017, *p* > 0.1) (*H3b* is not supported). In wave 2, individuals' decisions on medical spending are much more critical and rational as the changes in perceived risk of the pandemic overtime play a pivotal role in the decision-making process instead of using the shortcut of herd behaviour or panic buying factors. As such, individuals with significant changes in risk perception of the pandemic are much more likely to change their level of spending on medical goods and services. Thus, *H5* is supported.

### 4.9 Demographic characteristics and medical spending

Regarding the effects of individual demographic characteristics on medical spending, we have observed some interesting results. Income is the only variable that significantly impacts medical spending (β = 0.077, *p* < 0.05). While in wave 2, education is the one to exert effects on medical spending but in the negative relationship (β = −0.100, *p* < 0.01). These results support the differences in formulating medical spending between the two waves of the pandemic. In wave 1, under the effects of herd behaviour and panic buying patterns, the individual financial resource will be the only constrain on how much to spend on medical goods and services. In contrast, education plays an important role in deciding spending when the decision-making process involves more cognitive exercises and rational risk assessment analysis. Moreover, higher education is related to less medical spending in wave 2.

## 5 Discussion

This study makes four main contributions. First, this study extended the literature on individual medical spending in the current COVID-19 pandemic. Researchers have paid a great deal of attention to evaluate the changes in healthcare cost and service use patterns on the aggregate level.^1^^,^^29^^,^^32^ However, not many studies about the motivations and drivers of medical spending in the COVID-19 pandemic on individual levels have been conducted. This study addresses this gap using the HBM framework to explore the key drivers for medical spending behaviours in the current health crisis. The statistical results suggest that HBM components in this study such as health-seeking behaviours, demographic factors, COVID-19 perceived risks could be used as meaningful drivers for explaining the individual medical spending level in the COVID-19 pandemic.

Second, this study also extended the HBM framework by incorporating the heuristic-systematic information processing theory^8^ into the framework. This theory combination is used to explain how the relationships between components in the HBM framework change over time following the dynamism of the COVID-19 pandemic. This study found that there were distinctive relationship patterns of factors in the HBM framework between the first and second waves of the COVID-19 pandemic. This study indicates that medical spending in the first wave of the pandemic is unaffected by the perceived risks of the COVID-19 pandemic; somewhat, it is primarily influenced by panic buying behaviours or people's health-related tendencies. It appears that during the initial wave of the epidemic, individuals raise their proportion of spending (on food, necessities, and medical goods) impulsively and without detailed examination or on a reasonable basis (perceived risks). This problem can be explained partly by the first wave's absence of the cognitive effort to catch the information; individuals adopt the heuristic that impacts decisions without exertion or self-awareness. As a result, the perceived risks in the first wave are likely to be highly skewed, and people's medical spending is not based on their risk judgments.

However, panic buying behaviour has no significant impact on medical spending in the second wave of the pandemic. Unlike the first wave, the second wave's perceived risk considerably influenced people's medical spending due to the mediating effects of the second wave's increased perceived risk over the first wave. Hence, it could be seen that an abnormal situation such as the COVID-19 pandemic requires time for people to adjust their perceptions to actually reflect the external environment. Moreover, income significantly influenced the medical spending in wave 1 of the pandemic, while, education level was the primary demographic factor affecting medical spending in wave 2. It could be seen that the decision in wave 2 required more cognitive efforts and higher information processing capability than in wave 1. These findings are consistent with results from research that examine the roles of information overload^21^ and trust^4^ at the beginning of the pandemic. They found that, in the early stage of the pandemic, heuristic information processing was the main way how people process information and formulate prevention behaviours. The heuristics could come from individuals' preferences of political stances^4^ or they are forced to use the heuristic because of the information overload that comes from too frequent online news and cognitive capacity.^21^

Third, this study also contributes to the literature about health-seeking behaviours in the current COVID-19 pandemic and provides empirical evidence about its effects on perceived risks and prevention behaviours. Previous literature focuses on how health-seeking behaviours changes under the effects of the COVID-19 pandemic,^33^^,^^45^ this study provides a new approach to explore the impacts of individuals' health-seeking behaviours impacts on pandemic perceived risk. Using the health-seeking behaviours as modifying factors within the HBM framework, this study found that their roles significantly changes in wave 2 of the pandemic compared to wave 1. In the first wave, the impacts of health-seeking behaviours were direct and significant only on medical spending but not on the pandemic perceived risk as suggested by the HBM framework. This expected relationship was only significant in the wave 2 of the pandemic. This observation could be explained by the use of more cognitive effort in the second wave of the pandemic so that individuals' experience and information gathered from the health-related behaviours such as health-seeking behaviours could be reflected better as important inputs in the process of formulating pandemic perceived risk. Even though, this finding highlights the important role of the established health-related behaviours to medical spending behaviours in an extreme situation, especially under panic buying pressure. The healthier habits people have, the less panic spending pattern they express in an extreme situation.

Finally, regarding the consumer behaviours on medical goods and services, this study found that panic buying is one of the key factors influencing medical spending in the early stage of the COVID-19 pandemic. This phenomenon is also confirmed by some studies during the current pandemic.^10^^,^^27^^,^^30^^,^^34^ It is found in this study that, panic buying fully mediates the impacts of the pandemic perceived risks on medical spending. This finding is consistent with findings of the impacts of panic buying that are mainly caused by exacerbating the anticipation of future scarcity, psychological distress, and threat sensitivity.^5^ Nevertheless, it is also found that in this study, panic buying is a temporary state and its effects disappear in the second wave of the pandemic when systematic information processing and more cognitive efforts were in place instead of heuristics one. As panic buying could cause a temporary shortage in the supply chain, this finding emphasizes the roles of government and institutions in providing and diffusing detailed quality information about the pandemic and seriously combating the fake news to eliminate irrational exuberance of fears and anxieties during the pandemic.

## 6 Conclusion

This study considers how medical spending behaviour has changed over time as the COVID-19 epidemic has progressed and identifies critical factors that influence medical spending behaviours during the current health crisis. Based on the health belief model (HBM) and the heuristic and systematic information processing, this study found significant impacts of the individual's health-seeking behaviour, pandemic perceived risk, panic buying and demographic factors on medical spending. Moreover, the effects of these sets of factors on medical spending behaviours are not static but dynamic and changing along with the evolution of the two waves of the COVID-19 pandemic. Findings from this study could inform governments and institutions with valuable insights into the key drivers of individuals' medical spending during different phases of the pandemic and help them to formulate policies to combat the pandemic more effectively.

## Declaration of Competing Interest

The authors declare that they have no known competing financial interests or personal relationships that could have appeared to influence the work reported in this paper.

## Figures and Tables

**Table:** 

*Your gender* 1. Male 2. Female	*Your age is in age range* 1. 18 to 35 2. 35 to 45 3. 45 to 60 4. above 60
*Your level of education* 1. Diploma or lower 2. Bachelor's degree 3. Postgraduate degree	*Your Marital status* 1. Mariage 2. Single
*Employment status* 1. Full time 2. Part-time 3. Students/Students 4. Retired 5. Housewife 6. Unemployed or others	*Your monthly income (million VND)* 1. <10 3. 10–20 4. 21–30 5. 31–50 6. >50

**Table:** 

*Seek medical consultation promptly when one felt sick* 1. A lot/necessary/urgent/required 2. Frequent/important 3. Regularly 4. Occasionally but not too much 5. Very little or no	*Comply with physician's advice* 1. A lot/necessary/urgent/required 2. Frequent/important 3. Regularly 4. Occasionally but not too much 5. Very little or no
*A healthier lifestyle, exercise for one h/week* 1. A lot/necessary/urgent/required 2. Frequent/important 3. Regularly 4. Occasionally but not too much 5. Very little or no	*Control body weight* 1. A lot/necessary/urgent/required 2. Frequent/important 3. Regularly 4. Occasionally but not too much 5. Very little or no
*Avoid consuming too much sweet food* 1. A lot/necessary/urgent/required 2. Frequent/important 3. Regularly 4. Occasionally but not too much 5. Very little or no	*Avoid consuming food rich in cholesterol* 1. A lot/necessary/urgent/required 2. Frequent/important 3. Regularly 4. Occasionally but not too much 5. Very little or no
*Consume health food products* 1. A lot/necessary/urgent/required 2. Frequent/important 3. Regularly 4. Occasionally but not too much 5. Very little or no	*Getting enough sleep* 1. A lot/necessary/urgent/required 2. Frequent/important 3. Regularly 4. Occasionally but not too much 5. Very little or no
*Searching health-related information* 1. A lot/necessary/urgent/required 2. Frequent/important 3. Regularly 4. Occasionally but not too much 5. Very little or no	*Willing to pay more than VND 1,500,000 per month for health/medical services* 1. Willing to pay much more than 2. Willing to pay the amount 3. Can only pay the amount for a certain period 4. Can pay at the amount but not longer 5. Can't pay the amount
*Willing to spend more than 15h per month on health/medical services* 1. Willing to spend more time than 2. Willing to spend the amount of time 3. Can only spend the amount of time for a certain period 4. Can spend the amount of time but not longer 5. Can't spend the amount of time	*Keep the living quarter hygenic* 1. A lot/necessary/urgent/required 2. Frequent/important 3. Regularly 4. Occasionally but not too much 5. Very little or no
*Maintain good personal hygiene* 1. A lot/necessary/urgent/required 2. Frequent/important 3. Regularly 4. Occasionally but not too much 5. Very little or no	*Wash hands frequently* 1. A lot/necessary/urgent/required 2. Frequent/important 3. Regularly 4. Occasionally but not too much 5. Very little or no
*Mask-wearing behaviour when having a cold, flu, illness, sore throat* 1. A lot/necessary/urgent/required 2. Frequent/important 3. Regularly 4. Occasionally but not too much 5. Very little or no	*Wear a mask when going out in public* 1. A lot/necessary/urgent/required 2. Frequent/important 3. Regularly 4. Occasionally but not too much 5. Very little or no
*Mask-wearing behaviour at home* 1. A lot/necessary/urgent/required 2. Frequent/important 3. Regularly 4. Occasionally but not too much 5. Very little or no	*Avoid risk behaviours* 1. A lot/necessary/urgent/required 2. Frequent/important 3. Regularly 4. Occasionally but not too much 5. Very little or no

**Table:** 

*During the lockdown, how does your spending on food increase more than usual?* 1. Not at all 2. Very slightly 3. Moderately 4. To a significant degree 5. Very significant	*During the lockdown, how does your spending on neccesity goods increase more than usual?* 1. Not at all 2. Very slightly 3. Moderately 4. To a significant degree 5. Very significant
*How does your medical spending increase from the start of the COVID-19 pandemic?* 1. Not at all 2. Very slightly 3. Moderately 4. To a significant degree 5. Very significant *3. COVID-19 Perceived risk* *If I did experience the COVID-19 virus, it would have a severe effect on me personally?* 1. Totally disagree 2. Disagree 3. Neutral 4. Agree 5. Totally agree	*How likely can the COVID-19 virus infect me?* 1. Definitely not likely 2. Somewhat not likely 3. Neutral 4. Somewhat likely 5. Very likely

**Table:** 

*How dangerous is the second wave of the COVID-19 pandemic compared to the first wave in your opinion?* 1. Significantly decrease 2. Decrease 3. No change 4. Increase 5. Significantly increase	*How does your medical spending increase from the start of the second wave of the COVID-19 pandemic?* 1. Not at all 2. Very slightly 3. Moderately 4. To a significant degree 5. Very significant

## Appendix A Survey Questionnaire

### Spending behaviours

^⁎^Note: All questions are replicated for the first and the second wave of the survey.
